# Why Some Mice Are Smarter than Others: The Impact of Bone Morphogenetic Protein Signaling on Cognition

**DOI:** 10.1523/ENEURO.0213-22.2022

**Published:** 2023-01-09

**Authors:** Jacqueline A. Bonds, Elif Tunc-Ozcan, Sara R. Dunlop, Radhika Rawat, Chian-Yu Peng, John A. Kessler

**Affiliations:** 1 Department of Neurology, Northwestern University Feinberg School of Medicine, Chicago, IL 60611; 2 Department of Anesthesiology, University of California San Diego, San Diego, CA 92161

**Keywords:** bone morphogenetic protein, cognition, noggin, novel object recognition

## Abstract

Inbred mice (C57Bl/6) display wide variability in performance on hippocampal-dependent cognitive tasks. Examination of microdissected dentate gyrus (DG) after cognitive testing showed a highly significant negative correlation between levels of bone morphogenetic protein (BMP) signaling and recognition memory. Cognitive performance decline during the aging process, and the degree of cognitive decline is strongly correlated with aging-related increases in BMP signaling. Further, cognitive performance was impaired when the BMP inhibitor, noggin, was knocked down in the DG. Infusion of noggin into the lateral ventricles enhanced DG-dependent cognition while BMP4 infusion led to significant impairments. Embryonic overexpression of noggin resulted in lifelong enhancement of recognition and spatial memory while overexpression of BMP4 resulted in lifelong impairment, substantiating the importance of differences in BMP signaling in wild-type mice. These findings indicate that performance in DG-dependent cognitive tasks is largely determined by differences in levels BMP signaling in the dentate gyrus.

## Significance Statement

Throughout life, the generation of new neurons in the brain, or neurogenesis, are known to be important for helping to maintain cognitive abilities. The current study describes a significant mechanism contributing to the changes in neurogenesis in aging and provides a specific molecular target for intervention for aging-related changes in cognitive function.

## Introduction

The precise cellular and molecular basis for variations in cognitive abilities are largely unknown. Cognitive ability has been associated with numerous genetic loci as well as with environmental experiences, and the interplay of biology and experience helps to shape brain function (Miller, 2010; Zhang and Meaney, 2010; Davies et al., 2011; Kail and Barnfield, 2011; Powledge, 2011; D.S. Moore, 2015; Polderman et al., 2015; Johnson et al., 2016; Deary et al., 2022). Variability in so many genetic and epigenetic factors that impact cognitive function have made it difficult to identify any single molecular locus that determines aspects of cognitive performance. The availability of inbred strains mice with virtually identical genetic backgrounds simplifies the task since behavioral differences will largely reflect the impact of the environment. There also are differences in the several brain regions and biochemical signals that are involved in different cognitive functions, and this also complicates identification of the molecular pathways that determine cognitive ability. However, discrete brain regions have been identified that mediate some specific cognitive functions. For example, recognition and spatial memory functions involve several brain regions (such as the perirhinal cortex, amygdala, and frontal cortical regions) but is largely dictated by the hippocampal dentate gyrus (DG) and these functions are dependent on its structural integrity (Gilbert et al., 2001; Clelland et al., 2009).

A unique feature of the hippocampal DG is the presence of neural stem cells (NSCs) that give rise to new neurons throughout an individual’s lifetime. Numerous studies have documented significant age-related declines in hippocampal neurogenesis, as well as declines in hippocampus-dependent cognition, particularly in recognition and spatial memory functions, in rodents (Braak and Braak, 1991; Bernal and Peterson, 2004; Bizon et al., 2004; Ming and Song, 2005; Zhao et al., 2006; Dupret et al., 2008; Morgenstern et al., 2008; Lazarov et al., 2010). The parallel reductions in both neurogenesis and cognition during aging suggest the possibility of a direct relationship. Given the degree to which cognitive abilities vary among individuals and decline at varying rates with aging, we sought to identify the mechanisms regulating these variations and to investigate the extent of the correlation between neurogenesis and cognition. With these considerations in mind, we developed an unbiased strategy in which we first examined cognitive functions in cohorts of inbred mice, with a specific focus on recognition memory, and then subsequently examined the brains of these mice to determine whether specific signaling molecules could be identified that directly correlated with cognitive ability.

A strong candidate signaling pathway was the bone morphogenetic protein (BMP) pathway because of the substantial amount of data supporting its role as a critical regulator of hippocampal neurogenesis and spatial memory function (Bond et al., 2012; Fares et al., 2019). Increased levels of BMP signaling suppress the generation of adult-born neurons in the DG and impair performance in hippocampus-dependent cognitive tests, as well as anxiety-like behaviors (Gobeske et al., 2009; Bond et al., 2014; Yousef et al., 2015; Meyers et al., 2016; Tunc-Ozcan et al., 2019, 2021). Specifically, levels of BMP4 have been shown by our group, and many others, to have robust changes with age in the hippocampus of mice (Yousef et al., 2015; Meyers et al., 2016; Díaz-Moreno et al., 2018). It has also been observed to increase in expression in the aging human brain and was thus chosen to be investigated in the current study (Meyers et al., 2016). The progressive aging-related alterations that BMP signaling undergoes could potentially correlate with the aging-related decline in cognition (Meyers et al., 2016).

Thus, the aim of the current study was to investigate whether differences in BMP signaling in the DG of inbred mice are correlated with the observed natural variations in cognitive behavior in adult and in aged mice. Examination of microdissected DG after cognitive testing showed a highly significant negative correlation between levels of BMP signaling and cognitive performance. Further, both cognitive performance and hippocampal neurogenesis declined during the aging process, and the degree of cognitive decline was highly correlated with aging-related increases in BMP signaling. Interventions that decreased or increased BMP signaling in both adult and aging mice led to lifelong changes in cognitive performance that correlated with the level of BMP signaling. Thus, performance in these hippocampus-dependent cognitive tasks is largely determined by the level of BMP signaling in the DG.

## Materials and Methods

### Animals

All animal procedures were approved by the Institutional Animal Care and Use Committee (IACUC). All experiments were performed in accordance with the Public Health Service Policy on Humane Care and Use of Laboratory Animals. Behavioral analysis was performed during the light cycle. Wild-type (C57Bl/6) male mice of various ages were purchased from The Jackson Laboratory. The creation of the NSE-noggin and NSE-BMP4 transgenic mice was described previously (Bonaguidi et al., 2008).

### Novel object recognition (NOR)

Novel object recognition (NOR) was performed over 4 d. On days 1 and 2, mice were placed in the empty arena (582.25 cm^2^) for 10 min of habituation. On day 3, mice were placed in the arena with two identical objects (familiarization) that were placed 25 cm apart (diagonally) and were allowed to explore the objects for 20 min. On day 4, mice were placed in the arena with a familiar object and a novel object and were allowed to explore the objects for 20 min. The novel object was placed on the nonpreferred side of the arena, as determined in the familiarization phase, to avoid side preference. Identical and novel objects were similar in size but differed in color and shape. Object exploration was measured using LimeLight3 Software by designating two zones (with one object in each) which tracked each animal. Percent exploration was calculated by measuring time spent exploring each object over 20 min on both familiarization and novel testing days. Animals that did not explore for at least 20 s were excluded from analysis. The discrimination index was calculated by subtracting the time spent with the familiar object from the time spent with the novel object and divided it by the sum of the time spent with both objects [(T_novel_ – T_familiar_)/(T_novel_ + T_familiar_)].

### Statistical analysis

Exploration time of the familiar versus novel object during the test phase was compared within each group and analyzed by paired Student’s *t* test, *p < 0.05. Discrimination index was compared between groups and analyzed by unpaired Student’s *t* test, *p < 0.05.

### Spontaneous alternation Y-maze

The spontaneous alternation Y-Maze test was performed by placing the mouse in an arena consisting of three radial arms which were 60 cm in length, 14 cm wide, and 18 cm in height. Mice were allowed to explore the maze for 8 min (Meyers et al., 2016). Spontaneous alternation was quantified as the percentage of entry triads (entries into each of the three arms consecutively) to the total number of possible triads.

### Stereotaxic injection

Direct hippocampal injections of lentivirus expressing a shRNA against noggin (Bond et al., 2014; Brooker et al., 2017) were performed using a mouse stereotaxic instrument (Stoelting Co), Quintessential Stereotaxic Injector (Stoelting Co), and a Hamilton microsyringe (5 μl, NEUROS Model, 33 g, Blunt). Mice were anesthetized via inhalation of isoflurane. Two small craniotomies were performed over the hippocampus of each hemisphere (coordinates relative to bregma: 2 mm posterior, 1.5 mm lateral, 1.9 mm ventral) and 2 μl of virus was injected bilaterally into the DG at a rate of 0.5 μl/min. Two weeks postinjection, levels of noggin protein were analyzed, and any mice lacking successful viral infection in the DG were excluded from the study.

### Ventricular infusions

Adult mice were anesthetized in isoflurane and incised to reveal the cranium from λ to the occipito-cranial junction. Using blunt dissection, a subcutaneous pocket to receive the implanted osmotic pump was made from the caudal aspect of the cranial incision toward the tail. Using stereotactic guidance, a 1 mm cranial defect was drilled −0.4 and 1 mm lateral to bregma. The dura was opened using a 25-guage needle and the Alzet brain infusion cannula was placed within the lateral ventricle. A micro-osmotic pump (Alzet #1002), preloaded with the noggin or BMP4 solutions and attached to the cannula with a 1 cm plastic catheter, was implanted into the subcutaneous pocket before insertion of the cannula into the ventricle. Each pump delivered noggin (R&D) at 50 ng/μl or BMP4 (R&D) at 30 ng/μl for 15 d at a rate of 0.25 μl/h. The concentrations and the rate of delivery were decided according to preliminary testing. Behavioral testing was performed on day 14 (NOR) and 15 (Y-maze) followed by killing.

### Bromodioxyuridine (BrdU) administration

BrdU (Sigma catalog #10280879001) was administered via four intraperitoneal injections at 50 mg/kg over the course of 8 h. Animals were killed 24 h after the last BrdU injection via transcardiac perfusion with cold PBS.

### Immunoblotting

Following transcardiac perfusion, the DG from one hemisphere was microdissected on ice and mechanically homogenized in T-Per extraction reagent (ThermoFisher Scientific, catalog #78510) containing Halt protease and phosphatase inhibitors (ThermoFisher Scientific, catalog #78420). Lysates were centrifuged at 10,000 rpm for 15 min at 4°C and supernatant collected for Western blot analysis. Primary antibodies used were: mouse anti-BMP4 (1:1000, UM500038, OriGene), rabbit anti-noggin (1:1000, AB5729, Millipore), rabbit anti-phospho-Smad1/5/8 (1:500, AB3848, Millipore), rabbit anti-Smad1/5/8 (1:1000, sc-6031, Santa Cruz), and mouse anti-GAPDH (1:4000, MAB374, Millipore). Blots were imaged using Azure Biosystems C600 imaging system and densitometry analysis performed using ImageJ. When appropriate, blots were stripped using stripping buffer (ThermoFisher Scientific, catalog #46430) following manufacturer’s protocol.

### Immunohistochemistry

Following transcardiac perfusion with cold PBS, one hemisphere was removed to be postfixed in 4% PFA for 2 h. Brains were transferred to 30% sucrose for 24 h before being embedded in OCT and stored at −8°C; 25-μm-thick sections were serially mounted onto Superfrost slides using a cryostat (Leica). Tissue sections were washed three times in PBS, blocked in 5% normal goat serum (Jackson ImmunoResearch) with 0.25% Triton X-100 and 0.3 m glycine in PBS for 1 h at room temperature (RT). Primary antibody was diluted in blocking buffer and incubated overnight at 4°C. Sections were washed three times in PBS and incubated for 1 h at RT with a fluorophore-conjugated secondary antibody and DAPI for nuclear staining. Following three final washes in PBS, cover glass was applied with ProLong Gold Antifade Reagent.

For BrdU detection, slides were incubated in 2N HCl for 20 min at RT before initial blocking step. Slides were quenched with 0.1 m sodium tetraborate, pH 8.5 twice for 10 min and washed in PBS three times for 5 min. Blocking and antibody incubation were then performed as described above.

### Imaging and cell quantification

Images were acquired using a Leica TCS SP5 Confocal Microscope. Z-stacks of five images at 40× magnification of areas of interest in the dentate gyrus (DG) were obtained with the step size set at 2 μl. Z-stacks (four per animal) of equal thickness and equivalent rostro-caudal position were quantified for each sample. Stereological cell counting was performed using ImageJ software. For per volume quantification, cell counts were normalized to the volume of the DG cell layer imaged.

## Results

### BMP signaling correlates with hippocampal-dependent behaviors in adult and aged mice

**Figure 1.**
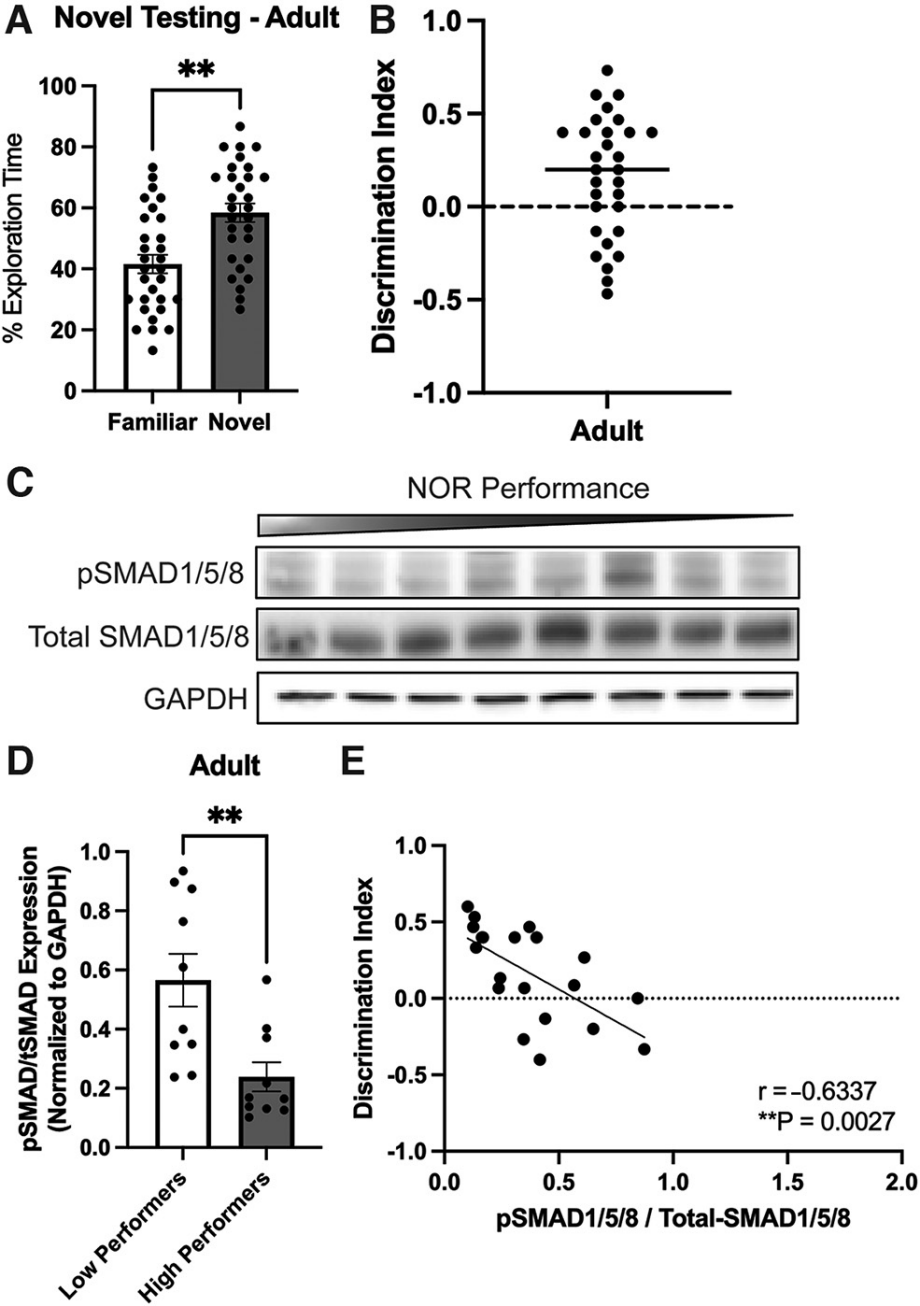
Decreased levels of BMP signaling correlates with improved recognition memory performance in adult mice. Long-term memory recall was probed using the novel object recognition (NOR) test. ***A***, Percentage of time exploring the novel object 24-h postfamiliarization was significantly increased in the adult mice (paired *t* test, **p < 0.01). ***B***, Discrimination index (DI) was calculated as (T_novel_ – T_Familiar_)/(T_Novel_ + T_Familiar_), where T = time. Animals with a discrimination index over 0.2 were considered a high performing animal. ***C***, ***D***, Western blot analysis of tissue lysate derived from the dentate gyrus of adult mice (low performers to high performers indicated by bar) show a significant decrease in levels of the ratio of pSMAD1/5/8 to total-SMAD1/5/8 in the high performers (DI > 0.2) compared with low performers (DI < 0.2; ***D***; unpaired *t* test, **p < 0.01). ***E***, When levels of BMP signaling are plotted against discrimination index, there is a significant negative correlation in both the adult and aged groups (Pearson’s *r* correlation, **p < 0.01).

To investigate the connections between natural variations in cognitive performance and BMP signaling in inbred mice, we first examined variations in recognition memory using the novel object recognition (NOR) test. NOR takes advantage of the natural bias of mice to explore novel stimuli and can be modified to probe each phase of memory formation from acquisition to consolidation to recall. This measure of cognition is largely dependent on the DG although it also involves perirhinal cortex (Suzuki, 1996; Wan et al., 1999; de Curtis and Paré, 2004; Balderas et al., 2013; S.J. Moore et al., 2013; Lueptow, 2017). During aging there is an impairment in the ability to convert short-term memories to long-term, thus we used a 24-h time point following familiarization to probe long-term memory recall. Our results show that mice aged six to seven months (adult) spend significantly more time exploring the novel object compared with the familiar object (Fig. 1*A*). Notably, however, there were substantial variations in performance among cohorts of inbred mice while still assuming a normal distribution. Two-thirds (66.7%) of the animals falls within 1 SD from the mean, while the remaining third (33.3%) of the animals show discrimination indexes that are within 2 SDs from the mean.

Based on the discrimination index measure of the NOR test, the animals were then separated into low performers and high performers using discrimination index of 0.2 as threshold that distinguish the two groups; Fig. 1*B*). We next asked the level of BMP signaling correlates with the variations in cognitive function among genetically identical mice. We thus examined the levels of canonical BMP signaling in the microdissected DG after performing the NOR test by measuring expression levels of phosphorylated and total SMAD1/5/8 proteins, which are the widely accepted readouts of BMP signaling (Wang et al., 2014; Nickel and Mueller, 2019). Comparing the levels of BMP signaling in the high and the low performing groups revealed a significant decrease in the ratio of pSMAD1/5/8 to total SMAD1/5/8 in the high performing animals (Fig. 1*C*,*D*). Further, recognition memory measured with the NOR test was highly negatively correlated (*r* = −0.6337, *p* = 0.0027) with the level of BMP signaling, measured as the ratio of pSMAD1/5/8 to total SMAD1/5/8 (Fig. 1*E*). These data support the correlative relationship between levels BMP signaling in the DG and cognitive behaviors associated with the DG.

**Figure 2.**
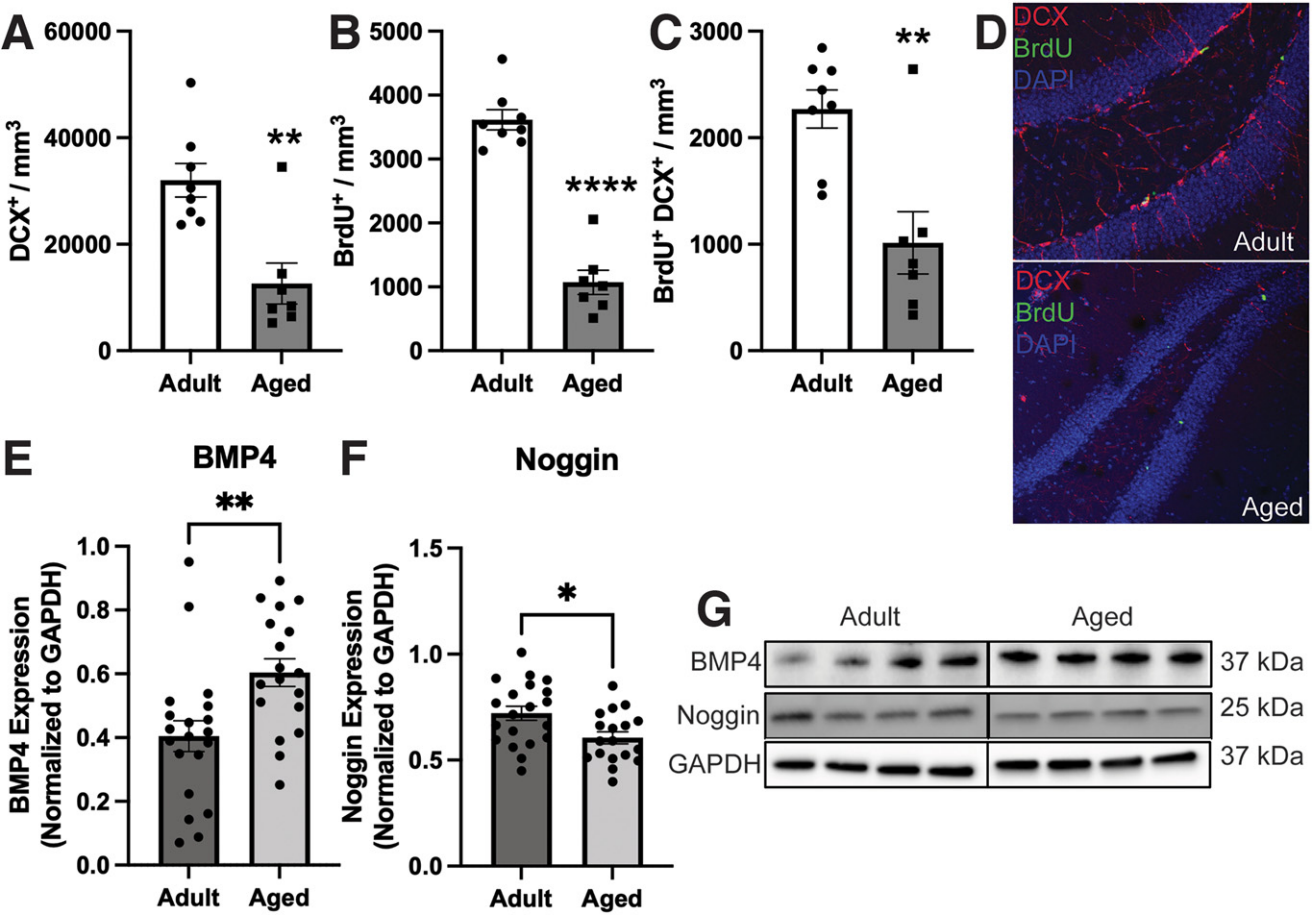
Alterations in neurogenesis and BMP signaling in adult versus aged mice. Analysis of adult-born neurons show that compared with adults (*N* = 8), the aged (*N* = 7) cohort displays a significant decrease in (***A***) DCX^+^, (***B***) BrdU^+^, and (***C***) BrdU^+^ DCX^+^ cell populations within the DG (representative images in ***D***; unpaired *t* test, ****p < 0.0001, **p < 0.01). Western blot analysis of microdissected DG tissue shows increased expression levels of BMP4 (***E***) were elevated in the aged compared with the adult, whereas the expression of noggin (***F***) was decreased in the aged compared with the adult (**p < 0.01, *p < 0.05, unpaired *t* test; *N* = 20, 18 for adult, aged). ***G***, Representative Western blot images.

**Figure 3.**
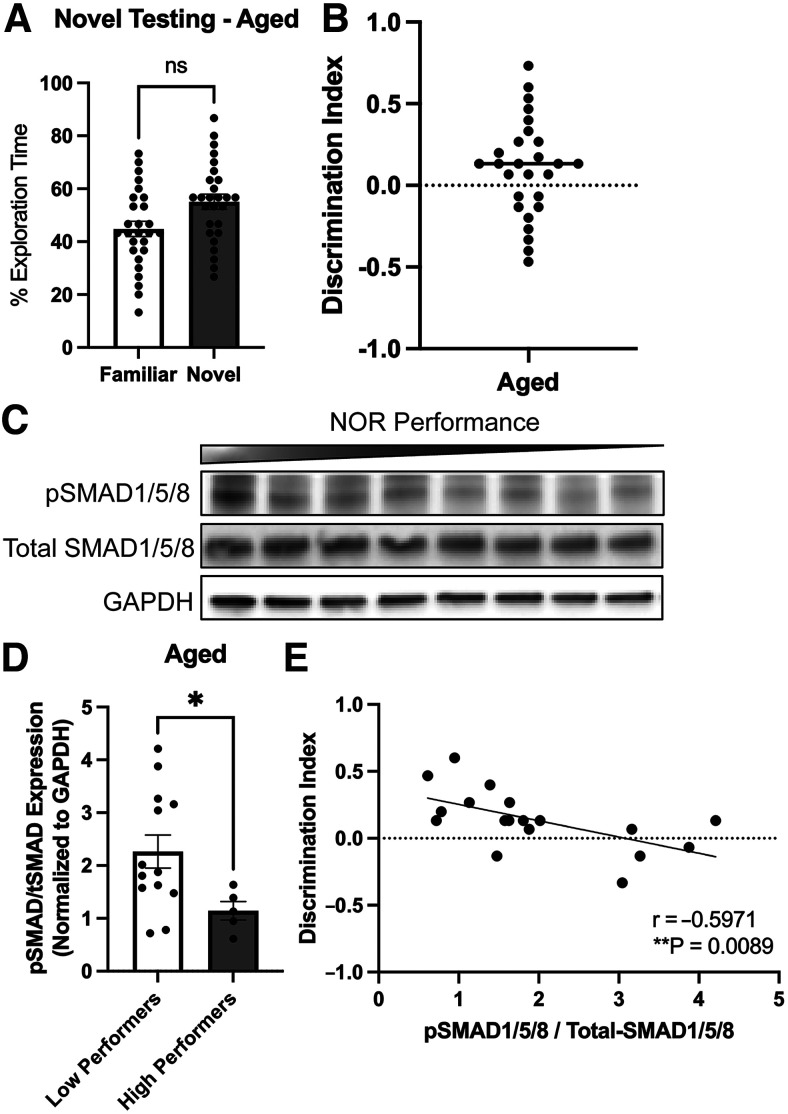
Decreased levels of BMP signaling correlates with improved recognition memory performance in aged mice. Long-term memory recall was probed using the novel object recognition (NOR) test. ***A***, Percentage of time exploring the novel object 24-h postfamiliarization was unchanged in the aged mice (paired *t* test, **p < 0.01). ***B***, Discrimination index (DI) was calculated as (T_novel_ – T_Familiar_)/(T_Novel_ + T_Familiar_), where T = time. Animals with a discrimination index over 0.2 were considered a high performing animal. ***C***, ***D***, Western blot analysis of tissue lysate derived from the dentate gyrus of aged mice (low performers to high performers indicated by bar) show a significant decrease in levels of the ratio of pSMAD1/5/8 to total-SMAD1/5/8 in the high performers (DI > 0.2) compared with low performers (DI < 0.2; ***D***; unpaired *t* test, *p < 0.05). ***E***, When levels of BMP signaling are plotted against discrimination index, there is a significant negative correlation (Pearson’s *r* correlation, **p < 0.01).

Many prior studies have shown that neurogenesis declines with age in the DG (Lazarov et al., 2010), and we similarly found that the number of newly generated neurons [doublecortin^+^ (DCX) cells; Fig. 2*A*] and the level of proliferation in the DG measured by incorporation of BrdU (Fig. 2*B–D*) both declined significantly in aged (17- to 18-month) compared with adult (five- to six-month) mice. Notably, levels of BMP4 expression were significantly elevated in the aged cohort, while levels of the BMP inhibitor, noggin, were decreased (Fig. 2*E–G*). Additionally, previous studies have observed that recognition memory declines with age, and we have shown that BMP signaling in the DG increases with age (Meyers et al., 2016). We therefore sought to determine whether cognitive performance in aged mice also correlates with the level of BMP signaling. As an overall group, 17- to 18-month-old mice displayed no significant difference in the NOR test between time spent exploring the novel versus familiar object (Fig. 3*A*,*B*), showing their decline in cognitive performance. However, when separated into high performers versus low performers, the level of BMP signaling was significantly higher in the low performing group (Fig. 3*C*,*D*). Further, there was a highly significant negative correlation (*r* = −0.6286, *p* = 0.003) between the level of BMP signaling and performance on the NOR test (Fig. 3*E*). Thus, memory recognition as measured by the NOR test depends on the level of BMP signaling in both adult and aged mice.

### Direct modulation of BMP signaling alters behavior and neurogenesis

**Figure 4.**
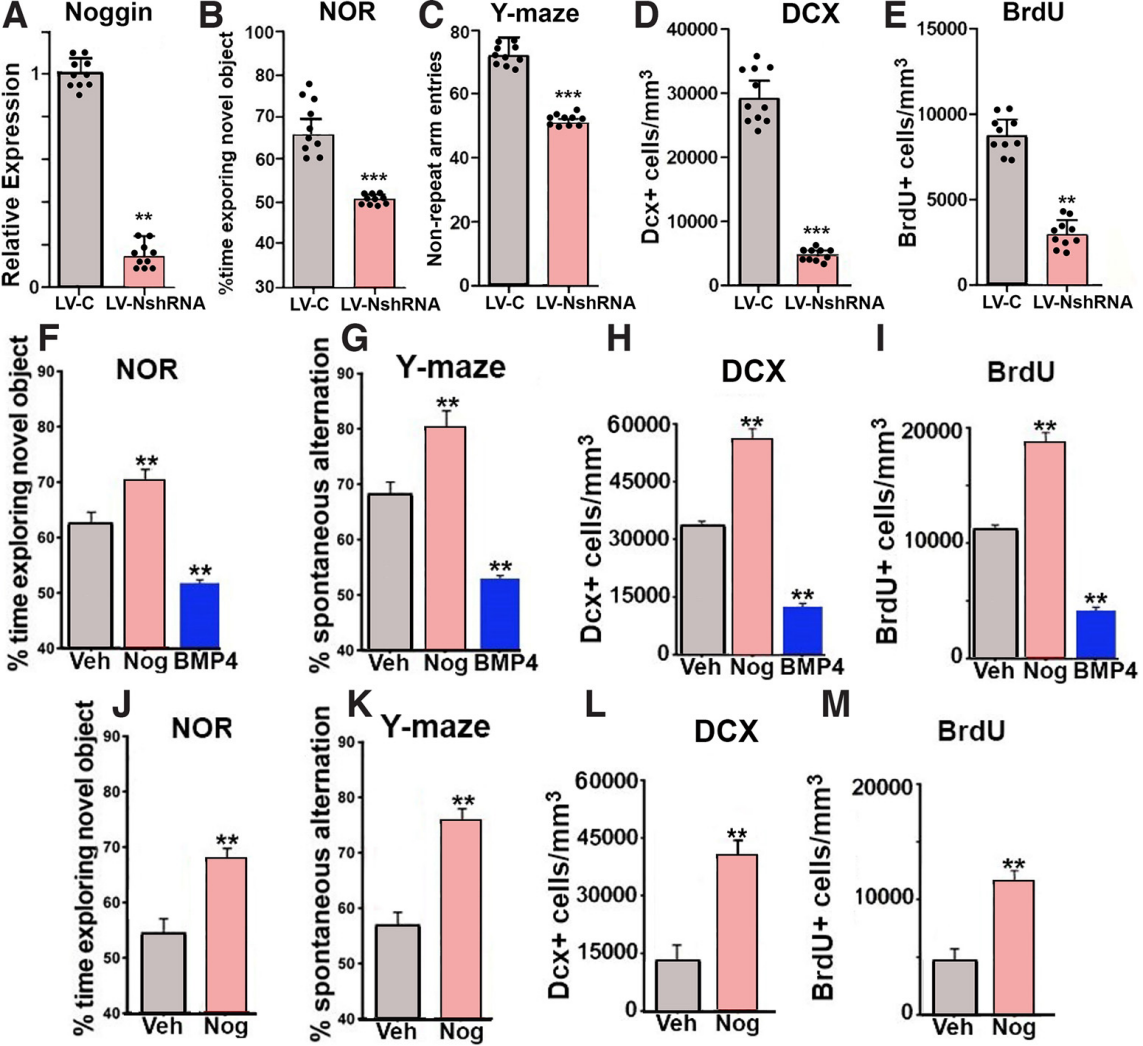
Decreased expression of the BMP inhibitor, noggin, in the dentate gyrus leads to a reduction of neurogenesis and impairments in recognition memory. ***A–E***, Control lentivirus (LV-C) or lentivirus expressing noggin shRNA (LV-NshRNA) was stereotactically injected into the DG of adult mice. ***A***, Noggin expression on day 15 is significantly reduced. ***B***, ***C***, Mice which received the LV-NshRNA displayed a significant decrease in the overall percentage of time spent exploring the novel object (***B***) and environment (Y-maze; ***C***). Quantification of (***D***) DCX^+^ and (***E***) BrdU^+^ cells within the DG on day 15 is significantly reduced. Infusion of noggin into the lateral ventricles of adult mice led to improvements in the NOR (***F***) and Y-maze (***G***) cognitive tests, as well as increased populations of DCX^+^ (***H***) and BrdU^+^ (***I***), while infusion of BMP4 had the opposite effect. Noggin infusion into the lateral ventricles of aged mice (12 month) showed improvements in the NOR (***J***) and Y-maze (***K***), as well as increased populations of DCX^+^ (***L***) and BrdU^+^ (***M***). For two-group analyses an unpaired *t* test was used (***A–E*** and ***J–M***). For three-group analyses, a one-way ANOVA with Tukey’s *post hoc* test was used (***F–I***); ***p < 0.005, *p < 0.05.

The findings above are all correlational and do not establish causality. We therefore used several different methods for altering levels of BMP signaling in the DG to establish a causal linkage. Based on our results that endogenous levels of BMP signaling are highly correlative with various aspects of DG-dependent cognition (Figs. 1, 2), our previously published data showing that BMP signaling regulates neurogenesis (Bond et al., 2014; Brooker et al., 2017), and our observation that age-related changes in BMP4 and noggin expression occur in parallel with changes in neurogenesis (Fig. 3), we investigated how altering BMP signaling impacted neurogenesis as well as cognition at various ages. First, a lentivirus expressing an shRNA against noggin was stereotactically delivered to the DG of two-month-old wild-type mice to knock-down noggin expression (Fig. 4*A*). These animals displayed impairment in the NOR test and the spontaneous alternating Y-Maze test which represents a measure of short-term spatial working memory (Fig. 4*B*,*C*). In support of our hypothesis, we also observed decreased populations of both DCX^+^ and BrdU^+^ cells (Fig. 4*D*,*E*) in the DG.

In a subsequent experiment noggin or BMP4 was infused into the lateral ventricles of two-month-old mice with subsequent evaluation of both behavior and neurogenesis. Ventricular infusion of noggin in two-month-old mice improved performance on both the NOR and Y-maze tests and increased the population of both DCX^+^ and BrdU^+^ cells (Fig. 4*F–I*). Conversely, infusion of BMP4 decreased these cell populations compared with vehicle control and decreased performance in both the Y-Maze and the NOR tests, as well as decreases in these specific cellular populations (Fig. 4*F–I*). Lastly, to assess the impact of inhibition of BMP signaling in aging animals where it is elevated compared with adults (i.e., middle-aged vs mature adult), noggin was infused into the lateral ventricles of 10-month-old mice. This resulted in improvements in the Y-Maze and the NOR test (Fig. 4*J*,*K*), as well as an increase in the populations of both DCX^+^ and BrdU^+^ cells (Fig. 4*L*,*M*), suggesting that acute inhibition of BMP signaling in aged mice may be an effective intervention for aging associated decline in DG-dependent cognition and neurogenesis.

**Figure 5.**
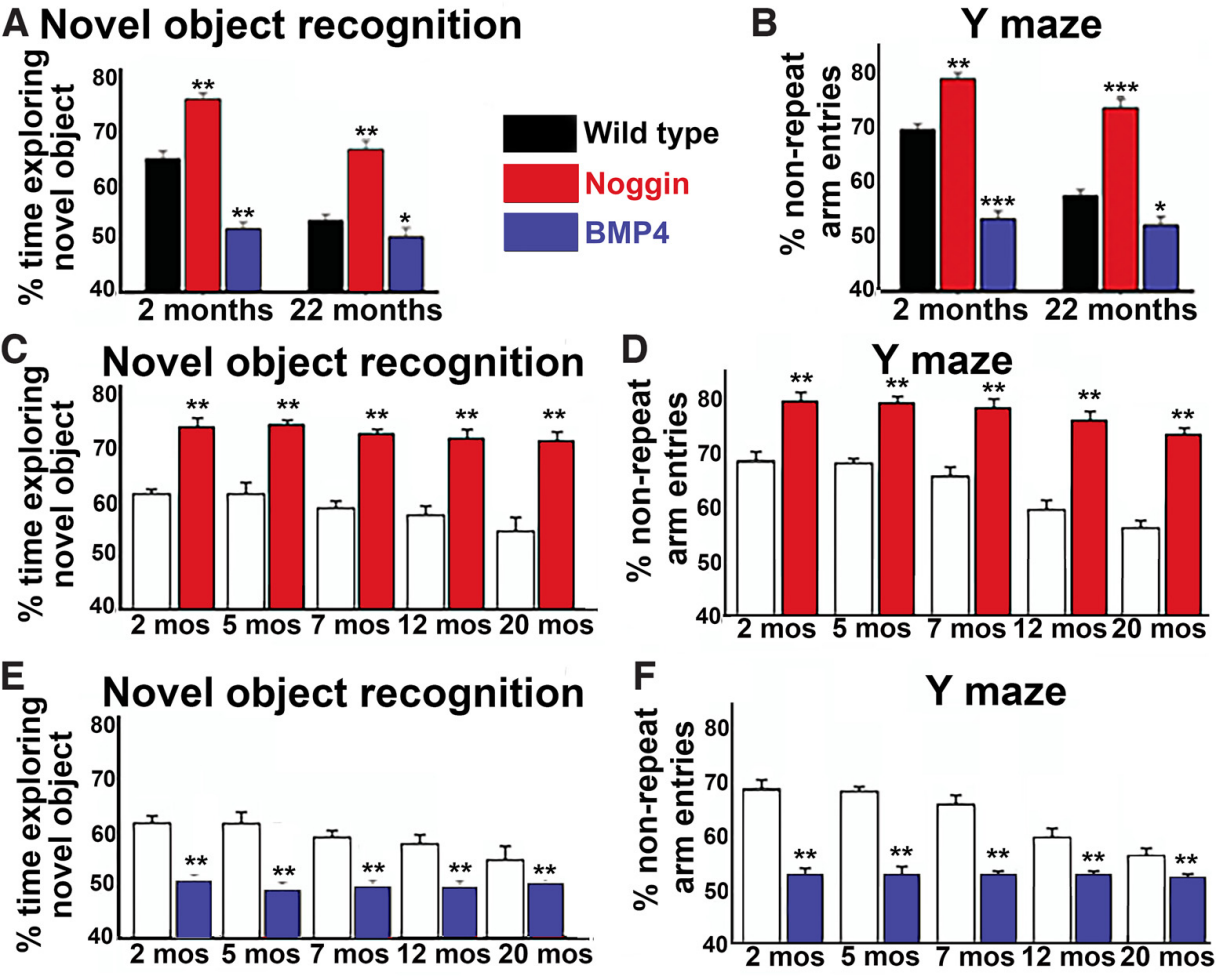
Levels of BMP signaling establish lifelong cognitive performance. NSE-Noggin, NSE-BMP4 animals and wild-type controls underwent NOR (***A***) and Y-maze (***B***) testing at two and 22 months of age. Inhibition of BMP signaling (NSE-Noggin) showed improvement of both recognition and spatial memory performance, whereas increased BMP signaling (NSE-BMP4) showed impairment in both these tasks. Cognitive performance was also probed at various timepoints (2, 5, 7, 12, and 20 months of age) in another cohort of mice, which shows either consistent improvement (***C***, ***D***, NSE-Noggin) or impairment (***E***, ***F***, NSE-BMP4). NOR was analyzed by paired *t* test (**p < 0.01) and Y-maze was analyzed by unpaired *t* test (**p < 0.01). For supporting data, please see Extended Data Figure 5-1.

To investigatethe impact of changes in BMP signaling over the lifetime of the animal, transgenic mice were generated that overexpress noggin or BMP4 under the neuron-specific enolase (NSE) promoter (Gobeske et al., 2009). As expected, noggin overexpression improved performance in both recognition (NOR) and spatial memory (Y-maze) tasks at both young and aged timepoints (Fig. 5*A*,*B*). Interestingly, 22-month-old mice with noggin overexpression performed significantly better in the Y-Maze and similarly in the NOR compared with two-month-old wild-type mice (Extended Data Fig. 5-1), supporting the critical role that BMP signaling regulation plays in cognition. Conversely, lifelong overexpression of BMP4 resulted in impairments in these tasks. In additional cohorts of mice, a more detailed temporal analysis of changes during aging (Fig. 5*C–F*) demonstrated a persistent, lifelong improvement in cognitive function in the noggin-overexpressing mice and lifelong decreases in cognitive function in BMP4-overexpressing mice. Overall, wild-type mice showed a progressive decrease with age in performance on both the NOR and Y-maze tests. Overexpression of noggin largely blunted this decrease in performance such that 20-month-old overexpressing mice performed better than two-month-old wild-type mice. BMP4 overexpressing mice showed little or no change in performance with aging but rather performed at a low level throughout their lifespan. In toto, these findings indicate that recognition and spatial memory performance are largely determined by the level of hippocampal BMP signaling.

10.1523/ENEURO.0213-22.2022.f5-1 Extended Data Figure 5-1 Noggin infusion improves cognitive performance in aged animals to similar levels as young animals. Infusion of noggin into the lateral ventricles of 22-month-old WT mice improves (***A***) Y-maze performance to above young WT (2-month-old) levels. ***B***, Preference for novel object is comparable between young WT and noggin-infused aged WT. Unpaired *t* test, *p < 0.05. Download Figure 5-1, TIF file.

## Discussion

Cognitive ability has been associated with numerous genetic loci as well as with environmental experiences, and the interplay of biology and experience helps to shape brain function (Miller, 2010; Zhang and Meaney, 2010; Davies et al., 2011; Kail and Barnfield, 2011; Powledge, 2011; D.S. Moore, 2015; Polderman et al., 2015; Johnson et al., 2016; Deary et al., 2022). To identify the impact of nongenetic factors, we used inbred mice with virtually identical genetic backgrounds so that variations among individuals in cognition reflect the impact of experiences rather than genetic differences. Cognitive functions decline to varying degrees during the normal aging process and several studies have implicated hippocampal neurogenesis as a determinant of cognitive performance (Lazarov and Marr, 2013). Additionally, there is a parallel decrease in hippocampal neurogenesis during aging and this process has been shown to be negatively regulated by the BMP signaling pathway which is increased during normal aging. Several prior studies have shown that this signaling pathway negatively regulates hippocampus-dependent cognitive functions as well as hippocampal neurogenesis (Bonaguidi et al., 2008; Gobeske et al., 2009; Bond et al., 2014; Yousef et al., 2015; Brooker et al., 2017; Tunc-Ozcan et al., 2019, 2021). Thus, the current study aimed to investigate whether varying levels of BMP signaling in the hippocampus could be used as a predictor of cognitive performance in the normal aging process.

The NOR test evaluates recognition memory and nonspatial learning, which involves detection of novelty that primarily, although not exclusively, involves the hippocampus (Lazarov et al., 2010; Bond et al., 2012, 2014; Meyers et al., 2016; Fares et al., 2019). There was a remarkable and highly significant negative correlation between the levels of BMP signaling, measured by levels of phosphorylated and total SMAD1/5/8 proteins, and performance on the NOR test in both adult and aged cohorts. Given that the performance of these cohorts assumed a normal distribution, these observations suggest that the endogenous level of BMP signaling in the DG is strongly correlated with recognition memory and nonspatial learning. Furthermore, we observed an age-related decline in neurogenesis which is paralleled by an increase in BMP4 expression and decrease in the expression of its main inhibitor, noggin. Taken together, we conclude that the aging related changes in BMP signaling may underlie the aging-related decline in this cognitive function.

To further investigate this, we directly increased levels of BMP signaling by infusing a lentiviral vector to silence noggin expression. This resulted in a decrease in levels of neurogenesis and impaired cognitive performance. Conversely, infusion of noggin into the ventricles improved cognitive function in both adult and aged mice, while BMP4 infusion achieved the opposite. These observations further confirmed the causal relationship between levels of BMP signaling and cognitive performance. While the present study does not establish a causal relationship between the changes in behavior and neurogenesis, our group recently showed a direct causal relationship between activity of newly generated granule neurons resulted from adult neurogenesis with depression-like behavior (Tunc-Ozcan et al., 2019). Future studies using this same methodology will be necessary to establish a similar causal relationship between neurogenesis and hippocampus-dependent cognitive behavior.

Cognitive functions decline with age in concert with many cellular and molecular changes in the brain including an increase in BMP signaling in the DG of both mice and humans (Bernal and Peterson 2004). We found a tight correlation between the levels of BMP signaling in the DG of aging mice and performance on the NOR test, suggesting that the aging-related increase in BMP signaling is responsible for the aging-related cognitive decline. Transgenic overexpression of noggin resulted in mice at very advanced ages (20−22 months) performed similarly to wild-type young adult mice (two to three months) in hippocampal-dependent behaviors tests. These observations strongly point to hippocampal BMP signaling as a determining factor of DG-dependent cognitive function; and that if it were to be inhibited, cognitive performance could be enhanced in the aging brain.

In summary, these findings indicate that recognition and spatial memory performance are strongly correlate with the level of BMP signaling in the DG, and that aging-related increases in BMP signaling in the DG play a role in the age-related declines in these cognitive functions. These data thus identify this molecular pathway as a target for the treatment of age-related cognitive decline.

## Synthesis

Reviewing Editor: Benjamin Deneen, Baylor College of Medicine Decisions are customarily a result of the Reviewing Editor and the peer reviewers coming together and discussing their recommendations until a consensus is reached. When revisions are invited, a fact-based synthesis statement explaining their decision and outlining what is needed to prepare a revision will be listed below. The following reviewer(s) agreed to reveal their identity: Ya Dong Li.

Overall, the reviewers felt this paper provides potentially important insights into why mouse behavior assays exhibit some degree of variability. While largely a correlative study, the findings were considered to be robust and well controlled, and will provide important insights that can be used by the field as a whole. Nevertheless, weaknesses were observed, including some questions about experimental use of NPR assays, some issues about pSMAD staining, and adult neurogenesis in aged mice. Also, some of the statements in the paper need to be toned down to more accurately reflect the results.
