# Supplementary figures and images for: Why Some Mice Are Smarter than Others: The Impact of Bone Morphogenetic Protein Signaling on Cognition
Source: eNeuro. 2023 Jan 9;10(1):ENEURO.0213-22.2022. doi: 10.1523/ENEURO.0213-22.2022 (PMC9833048; doi:10.1523/ENEURO.0213-22.2022)

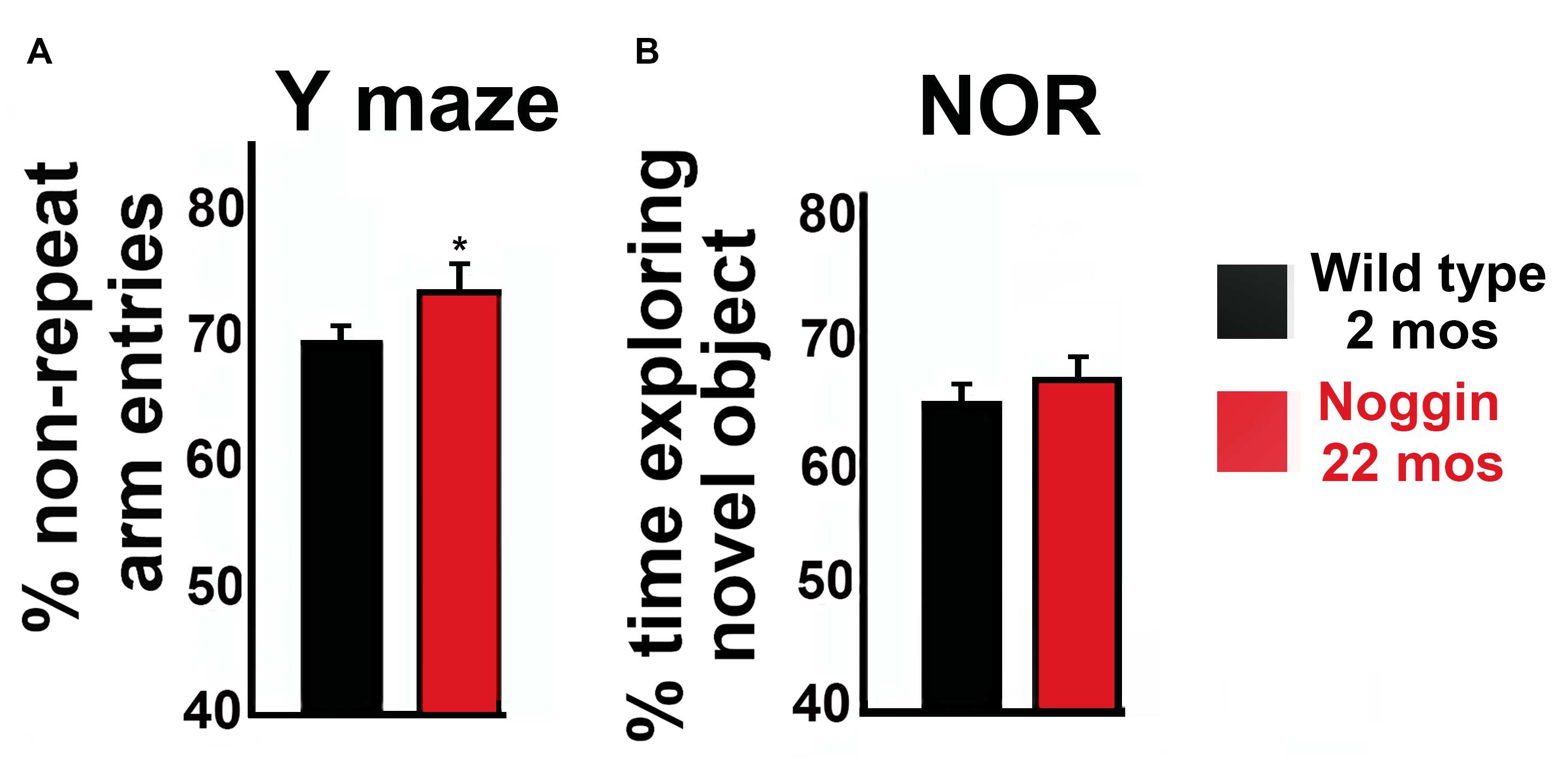

Supplement: Extended Data Figure 5-1 — Noggin infusion improves cognitive performance in aged animals to similar levels as young animals. Infusion of noggin into the lateral ventricles of 22-month-old WT mice improves (A) Y-maze performance to above young WT (2-month-old) levels. B, Preference for novel object is comparable between young WT and noggin-infused aged WT. Unpaired t test, *p < 0.05. Download Figure 5-1, TIF file. [file enu-eN-NWR-0213-22-s02.tif]
